# Interaction of Di-2-pyridylketone 2-pyridine Carboxylic Acid Hydrazone and Its Copper Complex with BSA: Effect on Antitumor Activity as Revealed by Spectroscopic Studies

**DOI:** 10.3390/molecules21050563

**Published:** 2016-04-28

**Authors:** Cuiping Li, Tengfei Huang, Yun Fu, Youxun Liu, Sufeng Zhou, Zhangyang Qi, Changzheng Li

**Affiliations:** 1 Department of Molecular Biology & Biochemistry, Xinxiang Medical University, Xinxiang 453003, Henan, China; lcp831220@163.com (C.L.); htengfei@yahoo.com (T.H.); fuyun9801@163.com (Y.F.); liuyouxun@126.com (Y.L.); sufengzhou@xxmu.edu.cn (S.Z.); qizhangyang0324@163.com (Z.Q.); 2 Henan Collaborative Innovation Center of Molecular Diagnostics and Laboratory Medicine, Xinxiang 453003, Henan, China

**Keywords:** di-2-pyridylketone 2-pyridine carboxylic acid hydrazone, copper complex, fluorescence, FRET, molecular docking

## Abstract

The drug, di-2-pyridylketone-2-pyridine carboxylic acid hydrazone (DPPCAH) and its copper complex (DPPCAH-Cu) exhibit significant antitumor activity. However, the mechanism of their pharmacological interaction with the biological molecule bovine serum albumin (BSA) remains poorly understood. The present study elucidates the interactions between the drug and BSA through MTT assays, spectroscopic methods and molecular docking analysis. Our results indicate that BSA could attenuate effect on the cytotoxicity of DPPCAH, but not DPPCAH-Cu. Data from fluorescence quenching measurements demonstrated that both DPPCAH and DPPCAH-Cu could bind to BSA, with a reversed effect on the environment of tryptophan residues in polarity. CD spectra revealed that the DPPCAH-Cu exerted a slightly stronger effect on the secondary structure of BSA than DPPCAH. The association constant of DPPCAH with BSA was greater than that of DPPCAH-Cu. Docking studies indicated that the binding of DPPCAH to BSA involved a greater number of hydrogen bonds compared to DPPCAH-Cu. The calculated distances between bound ligands and tryptophans in BSA were in agreement with fluorescence resonance energy transfer results. Thus, the binding affinity of the drug (DPPCAH or DPPCAH-Cu) with BSA partially contributes to its antitumor activity; the greater the drug affinity is to BSA, the less is its antitumor activity.

## 1. Introduction

Neoplastic cells need higher concentrations of iron and copper for their growth than normal cells [1,2], so the development of novel Fe and Cu chelators has become a promising anticancer strategy owing to their ability to inhibit cancer cell proliferation [3]. Accordingly, triapine, an iron chelator, is currently in phase II clinical trials [4]. Recently, a Dp44mT analog, di-2-pyridylketone-2-pyridine carboxylic acid hydrazone (DPPCAH) showed markedly antitumor activity against numerous cancer cell lines. This antitumor mechanism was mediated by its ability to inhibit topoisomerase, leading to cell cycle arrest and induction of DNA fragmentation [5]. Its copper complex, (DPPCAH-Cu) exhibited similar but stronger antitumor activity as indicated by several studies [6,7]. Many anticancer drugs have potent metal chelating ability. These metal chelators, especially copper chelates, possess enhanced biological activity compared to the non-chelating drugs. Mechanistic studies have revealed that the difference is at least partially related to the redox features of the copper complexes [5,8,9]. However, information related to the effect of the interactions of metal chelating agents with biological molecules, such as human serum albumin (HSA) or bovine serum albumin (BSA) and DNA and their contribution to cytotoxicity has received limited attention. DNA and proteins (especially enzymes) are of particular interest as targets for a wide range of anticancer and antibiotic drugs [10]. To understand the nature, structure and behavior of drugs in biological systems, studies related to drug interactions with proteins and DNA are required.

The biological activity of any drug is influenced by drug-protein interactions. Proteins, mainly HSA and bovine serum albumin (BSA) are extensively studied examples, as they are the most important carriers for a broad spectrum of exogenous and endogenous ligands. HSA-drug(s) interactions greatly influence the absorption, distribution, metabolism and excretion of drugs [11,12]. Recently, Merlot *et al.*, reported the cellular uptake of Dp44mT as a novel mechanism facilitated by human serum albumin (HSA) [13]. Accumulation of albumin occurs within the interstitium of solid tumors [14,15,16]. Chemically, BSA is a heart-shaped globular protein, containing three homologous domains (I, II, and III), and each domain includes, two sub-domains (IA and IB) [17]. BSA contains two tryptophan (Trp) residues, one (Trp134) is located on the surface of subdomain IB, and Trp213 located within the hydrophobic binding pocket of subdomain IIA. The interaction of BSA with endogenous and exogenous ligands mostly occurs in these domains.

The objective of the present study was to find the specific binding domains of BSA and DPPCAH by employing computer-aided molecular docking and spectral techniques. Numerous methods are available for investigating protein-ligand binding, such as equilibrium dialysis, fluorescence, calorimetry, and nuclear magnetic resonance [18], yet spectral techniques are widely used in determining the ligand and biomacromolecule interactions, hence our choice. Special emphasis is placed on determining the binding affinity of the DPPCAH and DPPCAH-Cu towards BSA, which could provide additional insight into the differences in antitumor activity detected between these ligands (with metal chelating ability) and their metal complexes.

## 2. Results and Discussion

### 2.1. BSA Mediated Cytotoxicity of DPPCAH and Its Copper Complex (DPPCAH-Cu)

**Figure 1:**
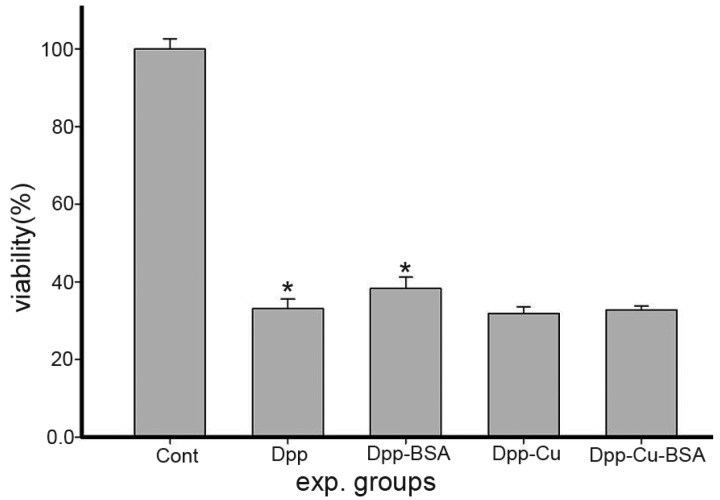
Inhibition of proliferation of HepG2 cells by DPPCAH and DPPCAH-Cu in the absence or presence of BSA. Dpp = DPPCAH; * *p* < 0.01 (*t*-test).

It has been reported that HSA could enhance the anti-proliferative activity of Dp44mT [13]. Thus, we examined the effect of BSA on both DPPCAH- and DPPCAH-Cu-mediated inhibition of HepG2 cells in which BSA was the main protein source in the cell culture. As shown in Figure 1, BSA attenuated the growth inhibition of HepG2 cells mediated by DPPCAH, but not DPPCAH-Cu, indicating that the effect of BSA on uptake or cytotoxicity of the drug was drug dependent, which was consistent with earlier reports [13].

Previously, we demonstrated that DPPCAH significantly inhibited the growth inhibition of HepG2 cells (IC_50_: 4.6 ± 0.2 μM), while DPPCAH-Cu significantly enhanced the antitumor activity [5]. In this regards, the weaker anti-proliferative activity of DPPCAH might have be due to the abatement of BSA. Hence, a study related to their interactions was necessary.

### 2.2. Interactions of DPPCAH and DPPCAH-Cu with BSA

**Figure 2:**
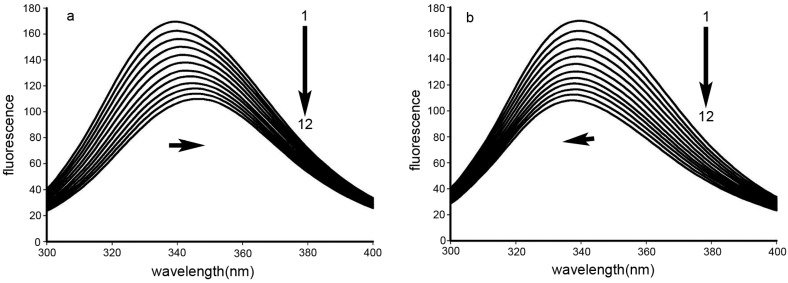
Stern–Volmer plots for the system (**a**) BSA-DPPCAH and (**b**) BSA-DPPCAH-Cu, C_BSA_ = 1.0 × 10^−5^ (M); pH = 7.2, at 288 K.

The two Trp residues of BSA are normally used as intrinsic fluorophores in spectral studies. Fluorescence quenching analyses of these Trp residues provides information regarding the interaction of the drug as quencher with BSA, revealing the mechanism(s) whereby small molecules bind to proteins. Figure 2 shows the fluorescence quenching spectra of BSA with varying concentrations of the DPPCAH and DPPCAH-Cu. The fluorescence intensities of BSA gradually decreased with the addition of the above drugs, suggesting that both DPPCAH and DPPCAH-Cu could associate with BSA. Notably, a bathochromic red shift occurred upon addition of DPPCAH (Figure 2a), unlike the blue shift induced by DPPCAH-Cu (Figure 2b), indicating that the investigated agents had different effects on the environment of Trp residues. The red shift specifies Trp residues exposed to the solvent, whereas the blue shift is a consequence of Trp residues located in a more hydrophobic environment [19].

### 2.3. Binding Sites and Binding Constants

**Figure 3:**
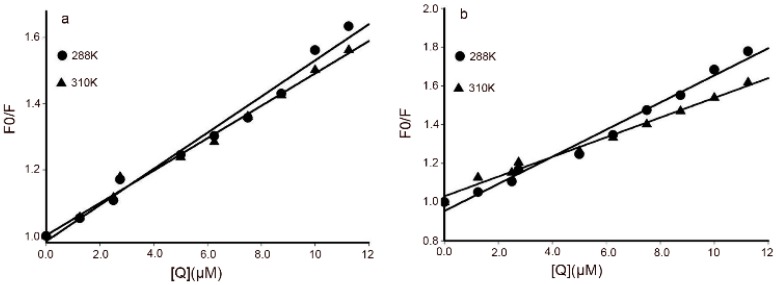
Stern–Volmer plots for BSA with (**a**) DPPCAH and (**b**) DPPCAH-Cu at 303 K and 310 K.

The observed fluorescence quenching and bathochromic shift (or blue shift) suggested interactions between DPPCAH and BSA or the possibility of formation of non-fluorescent complex(es). To investigate the possible mechanism responsible for fluorescence quenching, Stern–Volmer plots were used to interpret the spectral data. The procedure of fluorescence quenching was first assumed a dynamic quenching process. The dynamic quenching constant *K_sv_* and the apparent biomolecular quenching rate constant *K_q_* were calculated using the Stern–Volmer equation:

(1) $$\frac{F_{0}}{F}=1+K_{sv}[Q]=1+K_{q}\tau_{0}[Q]$$

where, *F_0_* and *F* are the steady-state fluorescence intensities in the absence and presence of a quencher, respectively; [*Q*] is the concentration of the quencher; *K_sv_* is the Stern–Volmer dynamic quenching rate constant; τ_0_ is the fluorescence lifetime of the protein without the quencher, the average life of a fluorescence molecule is 10^−8^ s; and *K*_q_ is the quenching rate constant of biomolecule. Based on the Stern–Volmer Equation (1), plots were generated, as shown in Figure 3. The plots exhibited good linear relationships, suggesting that a single type of quenching phenomenon, either static or dynamic quenching occurred during the formation of BSA-DPPCAH (or BSA-DPPCAH-Cu). To distinguish the quenching model, additional experiments were conducted at different temperatures, as dynamic and static quenching have different temperature dependencies. With an increase in temperature, the *K_sv_* values decrease for static quenching, and increase for dynamic quenching [20]. The data obtained at 303 K and 310 K were plotted (Figure 3a,b), and the slopes decreased (*K_sv_* from 7.0 × 10^4^ to 5.08 × 10^4^ for BSA-DPPCAH, 5.47 × 10^4^ to 4.9 × 10^4^ for BSA-DPPCAH-Cu) with increasing temperature, suggesting that DPPCAH and DPPCAH-Cu quenched the intrinsic fluorescence of BSA mainly by static quenching. *K_q_* values calculated for DPPCAH and DPPCAH-Cu ranged from 5 × 10^12^~7 × 10^12^, and >2 × 10^10^ L·mol^−1^·s^−1^, respectively, which further supported the static quenching model [21].

### 2.4. Binding Sites and Binding Constants

**Figure 4:**
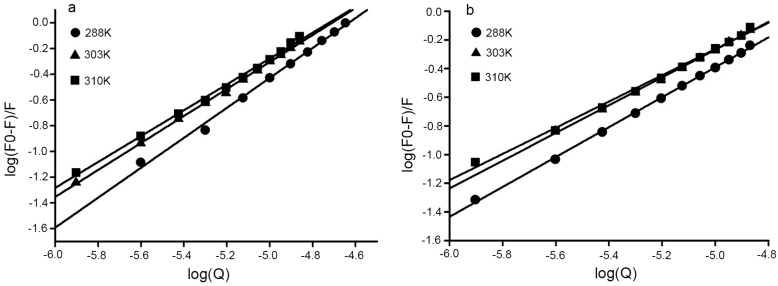
Plots of log [(F_0_ − F)/F] *vs.* log [Q] for (**a**) BSA-DPPCAH and (**b**) BSA-DPPCAH-Cu systems at three different temperatures; C_BSA_ = 10 μM; pH = 7.2 (10 mM Tris-HCl).

For the static quenching model, important binding parameters including association constant (*K_a_*) and number of binding sites (*n*) were calculated from the plots [22] using the equation:

(2) $$\log(\frac{F_{0}-F}{F_{0}})=\log\;K_{a}+n\log[Q]$$

where, *K_a_* is the binding constant, and n is the number of binding sites per BSA. *K_a_* and *n* were obtained from the intercept and slope of the curve. As shown in Figure 4a,b, the values of *K_a_* for DPPCAH and DPPCAH-Cu at 288 K were 1.35 ×10^5^ and 6.56 ×10^4^, respectively. The number of binding sites *n* was 1.0418 for DPPCAH and 1.1053 for DPPCAH-Cu, or approximately 1. Thus, the association of one molecule of quencher with one molecule of protein was confirmed. The value also indicated that a slight decrease of *K_a_* with an increase in temperature, which was in accordance with the aforementioned trend of *K_sv_*. Thus, these results indicate the formation of an unstable complex during the binding. Similar results were reported from studies analyzing interaction of eupatorin with BSA [17].

### 2.5. Binding Forces

**Figure 5:**
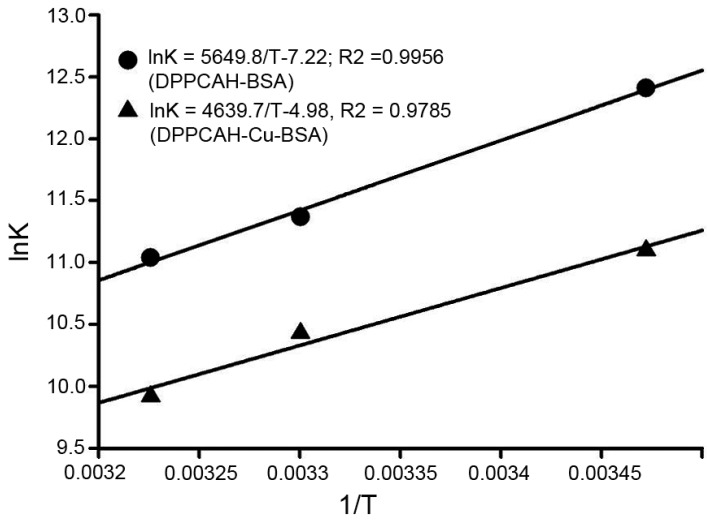
Van’t Hoff plot indicating the interaction between BSA and DPPCAH and DPPCAH-Cu; (pH 7.2). C_BSA_ = 10 μΜ; (pH 7.2).

The interaction of small molecules with biomacromolecule involves hydrogen bonds, electrostatic interactions, van der Waals, and hydrophobic forces. In the present study, the nature of the interaction involved in binding was determined based on thermodynamic parameters, namely, Δ𝐻 > 0 and Δ𝑆 > 0 would imply a hydrophobic interaction, Δ𝐻 < 0 and Δ𝑆 < 0 hint imply hydrogen bonding and van der Waals interactions, and Δ𝐻< 0 and Δ𝑆 > 0 imply electrostatic force interactions [23,24]. The thermodynamic parameters were obtained using the van’t Hoff Equation (3):

(3) $$\ln K=\frac{-\Delta H}{RT}+\frac{\Delta S}{R}$$

where, 𝐾 is the binding constant; *T* is the absolute temperature; and 𝑅 is the universal gas constant. Δ*H* and Δ*S* were obtained from the slope and intercept of the linear van’t Hoff plot (shown in Figure 5). The free energy change (Δ*G*) was estimated using the following formula:

Δ*G* = Δ*H* − *T*Δ*S*

Values of Δ*H*, Δ*S*, and Δ*G* for DPPCAH and DPPCAH-Cu binding to BSA are listed in Table 1. The negative value of Δ*G* reveals that the interaction process was spontaneous.

### 2.6. Energy Transfer between DPPCAH (DPPCAH-Cu) and BSA

**Figure 6:**
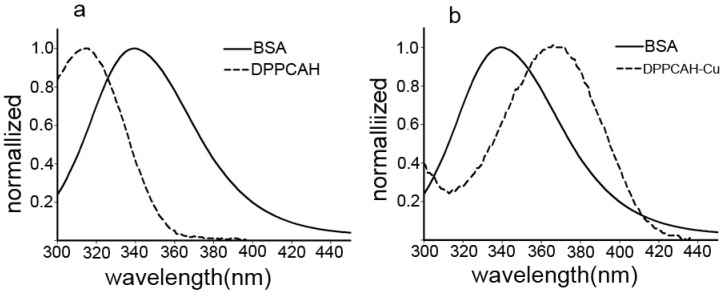
The overlap of the fluorescence spectra of BSA and the absorption spectra of (**a**) DPPCAH and (**b**) DPPCAH-Cu.

Our experimental data until now proved that DPPCAH and DPPCAH-Cu were bound to BSA in a 1:1 molar ratio. Further, the distance R between the two Trp residues in BSA and the bound DPPCAH (DPPCAH-Cu) was determined using the fluorescence resonance energy transfer (FRET) method [25]. Generally, FRET occurs whenever the emission spectrum of a fluorophore (donor) overlaps with the absorption spectrum of the bound molecule (acceptor). The efficiency of energy transfer could be used to evaluate the distance between the drug and the Trp213 residue in BSA. The relationship between the distance and efficiency of FRET is described by the following equation:

(4) $$E=1-\frac{F}{F_{0}}=\frac{R_{0}^{6}}{R_{0}^{6}+r^{6}}$$

where, r is the distance between acceptor and donor and, *R*_0_ is the critical distance when the energy transfer efficiency is 50%. The value of *R*_0_ is calculated using the following equation:

(5) $$R=8.8\times 10^{-25}\;K^{2}\;N^{-4}\Phi J$$

where, *K^2^* is the relative orientation of the transition dipoles of donor and acceptor (*K^2^* = 2/3 for a random orientation); *N* is the average refractive index of the medium (*N* = 1.336); ф is the fluorescence quantum yield of donor (ф = 0.15) [26]; *J* is a factor describing the spectral overlap between the emission spectrum of donor and the absorption spectrum of acceptor. *J* is calculated using the following equation:

(6) $$J=\frac{\sum F(\lambda )\varepsilon (\lambda )\lambda^{4}\Delta \lambda }{\sum F(\lambda )\Delta \lambda }$$

where, λ is wavelength. To calculate the distance (*r*), *E* and *J* value are required. These values are derived from the fluorescence data and overlapped area between the emission spectrum of BSA and the absorption spectrum of DPPCAH (or DPPCAH-Cu) as shown in Figure 6a (or Figure 6b) after normalization. The experimental value for the efficiency to be used in Equation (4) was estimated by measuring the fluorescence at equal protein-ligand concentration as described in [27]. Using Equations (4)–(6), *J*, *R*_0_ (nm), *E*, and *r* (nm) were calculated and are listed in Table 2.

The Trp residues of BSA involved in binding could be either Trp-134 or Trp-213. Trp-134 is located on the surface of the albumin molecule and is more exposed to a hydrophilic environment, whereas Trp-213 is buried deeply in a hydrophobic pocket of the protein [17,28]. Therefore, FRET was used to determine the distance between Trp-213 and the bound agents. The *r* values represent the distance from DPPCAH (and DPPCAH-Cu) to Trp-213 residue of BSA, and was 2.03 nm (*R*_0_ = 2.35 nm) and 2.32 nm (*R*_0_ = 2.7 nm), respectively. The calculated distance between BSA and DPPCAH was less than 7 nm and within 0.5*R*_0_ < r < 1.5*R*_0_, indicating that there was a high possibility of energy transfer from Trp-213 to DPPCAH (or DPPCAH-Cu) [29,30]. The calculated results were well predicted by Föster’s non-radioactive energy transfer theory [31], and are in accordance with the occurrence of a static quenching mechanism. These results might further verify that DPPCAH or DPPCAH-Cu were likely to be located in domain IIIA of BSA [32].

### 2.7. Conformational Investigation by Synchronous Fluorescence

**Figure 7:**
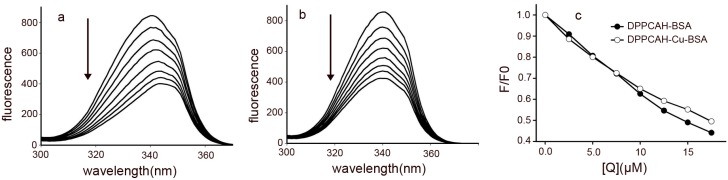
Effect of the addition of (**a**) DPPCAH and (**b**) DPPCAH-Cu on the synchronous fluorescence spectrum of BSA (Δλ = 60 nm); (**c**) changes in fluorescence quenched by agents: C_BSA_ = 10.0 μM, at 288 K.

The bathochromic (or blue) shift noticed during fluorescence titration of BSA with the reagents, implied potent changes in the environment of Trp residues. To confirm these environmental changes, synchronous fluorescence spectra were recorded, because this technique yields information pertaining to the change in the polarity of the microregion near fluorophore molecules. By using different *D*-value (Δλ) between the excitation and emission wavelength, fluorescence contributors from different aromatic amino acids in the protein can be identified. When Δλ is stabilized at 60 nm, synchronous fluorescence spectroscopy provides the characteristic information specific to Trp residues [33]. When λ_em_ exhibits a blue shift, it indicates a decreased microenvironment polarity and increased hydrophobicity of Trp residues, while a red shift indicates an increased microenvironment polarity and decreased hydrophobicity [25,34]. The synchronous fluorescence spectra of the DPPCAH and DPPCAH-Cu at Δλ = 60 are shown in Figure 7. The maximum emission wavelength of Trp had a significant red shift upon addition of DPPCAH (Figure 7a). Accordingly, the quenching of fluorescence intensity of Trp residue was decreased by 55% (Figure 7c), indicating that DPPCAH reached subdomain IIA [35], causing a change in polarity around the Trp residue. In contrast, the addition of DPPCAH-Cu led to a blue shift in the emission wavelength of Trp (Figure 7b) with a subsequent decrease in fluorescence intensity by 50% (Figure 7b,c), suggesting an increase in the hydrophobicity of the Trp environment [36]. Although DPPCAH and DPPCAH-Cu could bind BSA, they induced different effects in BSA.

### 2.8. Circular Dichroism Spectra

Circular dichroism (CD) spectroscopy is a useful technique for ascertaining the possible influence of a drug on the secondary structure of proteins. CD measurements were performed in the absence or presence of different concentrations of DPPCAH (or DPPCAH-Cu). The presence of two negative bands at 208 and 222 nm in the ultraviolet region was observed as reported earlier [37,38].

**Figure 8:**
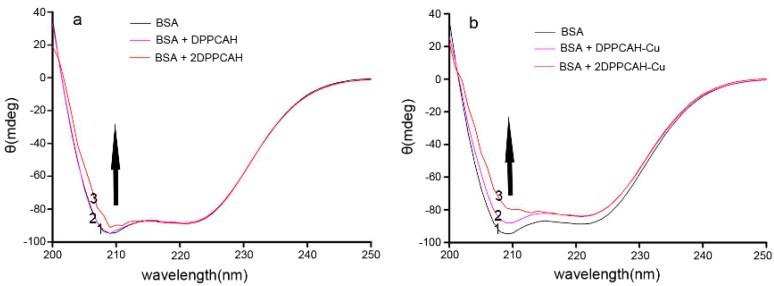
The CD spectra of (**a**) DPPCAH-BSA and (**b**) DPPCAH-Cu-BSA system: DPPCAH (or DPPCAH-Cu) concentrations were 0.0, 15, and 30 μM from 1 to 3, respectively. C_BSA_ = 15 μM, pH = 7.4, and T = 288 K.

As shown in Figure 8, the addition of DPPCAH (Figure 8a) and DPPCAH-Cu (Figure 8b) caused a decrease in bands, but the effect of DPPCAH-Cu was stronger. The results suggest an alteration in the secondary structure of BSA in the presence of the agents. A direct calculation of secondary structures using experimental data was employed [17,37], but owing to the larger errors, the K2D2 software was used to simulate the experimental data of CD in the present study [39]. The simulated curves are represented in the Supplementary Materials (Figure S1). In the absence of DPPCAH, the α-helix and β-sheet of BSA were 52% and 8.29% respectively. Following addition of DPPCAH or DPPCAH-Cu, only the latter led to a 3.5% decrease in α-helix, 1.45% increase in β-sheet at the molar ratio of drug/protein (d/p) = 2.

### 2.9. Molecular Docking

**Figure 9:**
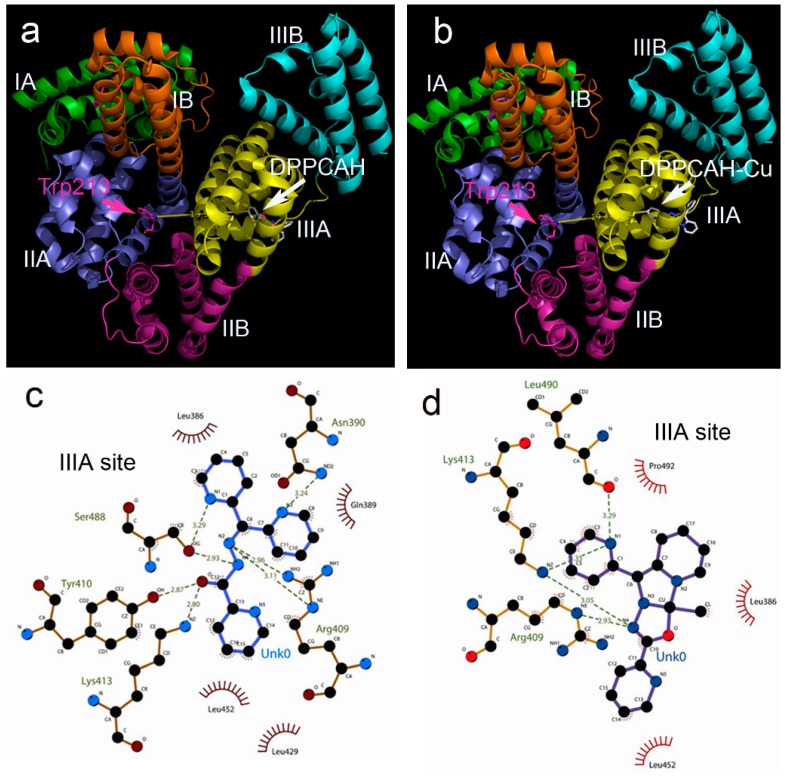
The interaction modes between of BSA with DPPCAH and DPPCAH-Cu: (**a**) DPPCAH was located in the IIIA site; (**b**) DPPCAH-Cu was located in the IIIA site; (**c**) interaction of DPPCAH with environment residues (in the figure Unk0 = DPPCAH); (**d**) interaction of DPPCAH-Cu with environment residues (in the figure Unk0 = DPPCAH-Cu). Yellow line indicates the distance (Figure 9a,b) and dark green dash lines (Figure 9c,d) indicated hydrogen bonds. The eye-like curved dark red lines represented the hydrophobic residues in contact.

The interaction between DPPCAH (DPPCAH-Cu) and BSA revealed by spectral data prompted us to probe the potential structural basis using molecular docking methods. Molecular docking is a widely used tool to predict the interaction of small molecules with biomolecules. The crystal structure of BSA (PDB ID: 4or0) was retrieved from RCSB Protein Data Bank. The DPPCAH and DPPCAH-Cu were individually docked into the subdomain IIIA of BSA. Their binding environments in BSA are shown in Figure 9.

The Simulating Affinity energies for DPPCAH and DPPCAH-Cu with BSA were −8.6 and −6.4 kcal/mol, respectively. These data were consistent with the data obtained from fluorescence quenching (−8.12 kcal/mol at 288 K for DPPCAH and −7.1 kcal/mol for DPPCAH-Cu, respectively). Values of theoretical distance between bound ligands and Trp-213 residue were measured using PyMol, and was found to be 1.95 (for DPPCAH in Figure 9a) and 1.94 nm (for DPPCAH-Cu in Figure 9b), which were close to the results obtained from FRET (2.03 nm and 2.32 nm). The interaction of DPPCAH/DPPCAH-Cu with the surrounding residues is shown in Figure 9c (or Figure 9d). A greater number of hydrogen bonds were formed for DPPCAH compared to DPPCAH -Cu (Figure 9c,d), which was consistent with the thermodynamics analyses results. Recently, the crystal structures of BSA with naproxen revealed that most of the binding sites present in serum albumin were interactions with more than one class of ligand [40]. We assumed a similar behavior for DPPCAH/DPPCAH-Cu as naproxen, and therefore DPPCAH and DPPCAH-Cu were docked at the IIA site (Sudlow’s site I). The resulting interactions with Trp213 and other residues are shown in Supplementary Materials (Figure S2). The introduction of DPPCAH into Sudlow’s site I decreased the distance of His241 and Arg198 to Trp213, which might have contributed to the bathochromic shift observed in the fluorescence titration. In contract to DPPCAH, the introduction of DPPCAH-Cu caused greater number of non-polar residues to approach Trp213, leading to the blue shift in fluorescence titration, suggesting an enhanced hydrophobic environment.

## 3. Materials and Methods

### 3.1. General Information

All the reagents and solvents used were of AR grade. The concentration of Ct-DNA in the stock solution was determined by UV using the molar absorption coefficient ε_260_ = 6600 L·mol^−1^·cm^−1^ at 260 nm.

### 3.2. Cytotoxicity Assay (MTT Assay)

A stock solution of DPPCAH (10 mM) was prepared in 80% DMSO, and was diluted to the required concentration with culture medium when used. The DPPCAH-Cu was prepared by mixing highly concentrated copper chloride with DPPCAH based on 1:1 molar ratio, and diluted to the required concentration with water [5]. BSA was dissolved in PBS and was sterilized by filter sterilization method. HepG2 cells were cultured in RPMI-1640 medium supplemented with 10% fetal calf serum (FCS) and antibiotics. The cells collected in the exponential-phase (5 × 10^3^ /mL) were seeded equivalently into a 96-well plate, followed by 6 h of incubation. The medium was then removed and the cells were washed with PBS. To the cells, 100 μL of medium without FCS along with varying amounts of DPPCAH or DPPCAH–BSA (1:1) (similarly for its copper complex) was added, followed by 8 h of incubation at 37 °C in a humidified atmosphere of 5% CO_2_. Further, 10 μL of MTT solution (5 mg/mL) was added to each well and incubated for 4 h. The medium was removed by aspiration and 100 μL of DMSO was added to each well in order to dissolve the formazan crystals. The plate was read using a microplate reader (MK3; Thermo Fisher Scientific (Shanghai) Co. LTD, Shanghai, China) at 570 nm and the measurement of absorbance represented an estimate of the number of viable cells. Percent growth inhibition was defined as percent absorbance inhibition within appropriate absorbance in each cell line. The assay was performed in triplicate.

### 3.3. UV–Vis Spectrophotometric Measurements

Absorbance spectrum of DPPCAH and DPPCAH-Cu were recorded on a UV-2450 spectrophotometer (Shimadzu (Suzhou) Co. Ltd., Suzhou, China) within the UV region in the range of 300 to 450 nm. Final concentrations of the agents concentration used for measurements were 10 μM (in Tris-HCl buffer, pH 7.2).

### 3.4. Fluorescence Measurements

For the fluorescence spectra, the excitation wavelength was set at 280 nm and the emission spectra were recorded in the range of 300 to 450 nm. Both excitation and emission slit widths were adjusted to 5 nm. In this assay, 4.0 mL of BSA solution at a fixed concentration of 10 × 10^−5^ M was accurately added into the quartz cell (1 cm path length), and was manually titrated by successive additions of 10 mM DPPCAH (or DPPCAH-Cu) at intervals of 5 min. Fluorescence emission spectra were then measured on a RF-5301 Spectrofluorophotometer (Shimadzu Scientific Instruments, Kyoto, Japan) at three different temperatures (288, 303, and 310 K). In order to calculate the peak area, the integration of spectra was done in the range of 300 to 450 nm. Synchronous fluorescence spectra were recorded by simultaneously scanning the excitation and emission monochromators, in which a constant wavelength interval was maintained between them. The wavelength interval (Δλ = λ_em_ − λ_ex_) was fixed individually at 60 nm, at which characteristic information of pertaining to Trp residues was obtained.

### 3.5. Measurements of CD Spectra

BSA CD spectra in the range of 190–250 nm were recorded on a Chirascan (Applied Photophysics Ltd., Surrey, UK) with a 1.0 cm quartz cell in nitrogen atmosphere. BSA CD measurements in the presence and absence of DPPCAH (or DPPCAH-Cu) were performed with a scan rate of 50 nm/min. The A stock solution of 1.5 × 10^−5^ M BSA was prepared in PBS. A 10 mM stock solution of DPPCAH was prepared in DMSO and the stock solution was diluted to 1.5 mM with water for measurements. Similarly, a solution of DPPCAH-Cu was prepared by mixing the DPPCAH solution with higher concentration of CuCl_2_ in water. The BSA concentration was held constant, while the DPPCAH concentration was varied (0, 1.5, 3.0 × 10^−5^ M). The final concentration of DMSO in the measurements was less 0.5%. The buffer solution was chosen as the blank, and was manually subtracted from each spectrum during scanning. Each sample was scanned three times and the average was recorded for the CD spectrum.

### 3.6. Molecular Docking

The AutoDock 4.0 software was used for the docking studies [41]. The crystal structure of BSA (PDB entry code 4or0) was downloaded from the RSCB Protein Data Bank (http://www.rscb.org), and the ligand structures were generated from ChemDraw. The AutoDock program was used to locate the appropriate binding orientations and ligand conformations in the BSA binding pocket. AutoDocktools was employed for creating PDBQT files from PDB files. AutoGrid program created the three-dimensional grid box. The grid box (22 × 24 × 28) was generated according to the naproxen (or grid box (38 × 44 × 54) was set according to the Trp 213 residue). It had a distance of ~20 Å to the ligand, which was similar to the result from our experimental data. The crystallographic conformation of the compound naproxen in BSA (PDB code 4or0) was utilized as a reference to evaluate the accuracy of the predicted model [42]. PyMol and LigPlot displayed the conformation, interaction and distances between bound ligands and the Trp213 residue around the binding cavity [43,44].

## 4. Conclusions

Drugs such as doxorubicin, ciprofloxacin, and mercaptopurine are potential metal chelators used in the clinic. Their potential chelating ability to form metal complexes was proved by both *in vitro* and *in vivo* studies. This metal chelating property of the drugs identify them as candidates with biological activity. In most cases, the cytotoxic data obtained from cell culture experiments indicated that the medium had to be supplemented with fetal calf serum to sustain cell growth. However, the effect of serum proteins on the cytotoxicity of drugs has received limited attention. BSA (HSA) is an important carrier of drugs, which also possesses the ability to interact with the drugs, altering their biological effect. In this study, the effect of BSA on the cytotoxicity of DPPCAH and DPPCAH-Cu was investigated. Our results indicate a drug dependent effect of BSA as it attenuated the antitumor activity of DPPCAH, but not of DPPCAH-Cu.

Recently, crystal structures of serum albumin revealed that its binding sites of BSA were able to interact with more than one class of ligands. DPPCAH and DPPCAH-Cu behave in a similar way as naproxen and bind to either IIA or IIIA site. DPPCAH makes the surrounding of Trp213 residue more polar, but DPPCAH-Cu makes the surroundings more hydrophobic. Thermodynamic parameters from fluorescence quenching experiments identified the Δ𝐺 of DPPCAH is to be more negative than that of DPPCAH-Cu. Accordingly, the association constant of DPPCAH was greater than that of DPPCAH-Cu, which was consistent with the molecular docking results. The introduction of DPPCAH led some polar amino acids being closer to Trp-213, but DPPCAH-Cu had an opposite effect, explaining the bathochromic (or blue) shift observed in fluorescence titration experiments. CD spectra results indicated less of an effect of DPPCAH on the secondary structure of BSA compared to DPPCAH-Cu, suggesting diverse mechanisms of action. In summary, this study provides valuable insight into the differences in the binding affinity of BSA to DPPCAH and DPPCAH-Cu that may be partially contributive to their antitumor activity.

## Figures and Tables

**Table 1:** The calculated values of Δ𝐻, Δ*S* and Δ*G* at 288 K.

System	Δ𝐻 (kJ·mol^−1^)	Δ*S* J/mol·K	Δ*G* (kJ·mol^−1^)
BSA-DPPCAH	−46.97	−60.0	−29.69
BSA-DPPCAH-Cu	−38.57	−41.5	−26.61

**Table 2:** The calculated values of *J*, *r*, *R*_0_, and *E* of BSA with DPPACH (or DPPACH-Cu).

System	*J* (cm^3^·L·mol^−1^)	*E* (%)	*R*_0_ (nm)	*r* (nm)
BSA-DPPCAH	7.424 × 10^13^	0.297	2.35	2.04
BSA-DPPCAH-Cu	1.699 × 10^14^	0.288	2.70	2.32

## Supplementary Materials

Supplementary materials can be accessed at: http://www.mdpi.com/1420-3049/21/5/563/s1.

## Abbreviations

The following abbreviations are used in this manuscript:

BSA	Bovine serum albumin
DPPCAH	Di-2-pyridylketone 2-pyridinecarboxylic acid hydrazone
DPPCAH-Cu	Copper complex of di-2-pyridylketone 2-pyridinecarboxylic acid hydrazone
FRET	Fluorescence resonance energy transfer
