# Supplementary material for: Interaction of Di-2-pyridylketone 2-pyridine Carboxylic Acid Hydrazone and Its Copper Complex with BSA: Effect on Antitumor Activity as Revealed by Spectroscopic Studies
Source: Molecules. 2016 Apr 28;21(5):563. doi: 10.3390/molecules21050563 (PMC6274005; doi:10.3390/molecules21050563)
Supplement: Supplementary file 1 [file molecules-21-00563-s001.pdf]

Supplementary Materials: Interaction of Di-2-pyridylketone 2-pyridine Carboxylic Acid Hydrazone and Its Copper Complex with BSA: Effect on Antitumor Activity as Revealed by Spectroscopic Studies

Cuiping Li, Tengfei Huang, Yun Fu, Youxun Liu, Sufeng Zhou, Zhangyang Qi and Changzheng Li

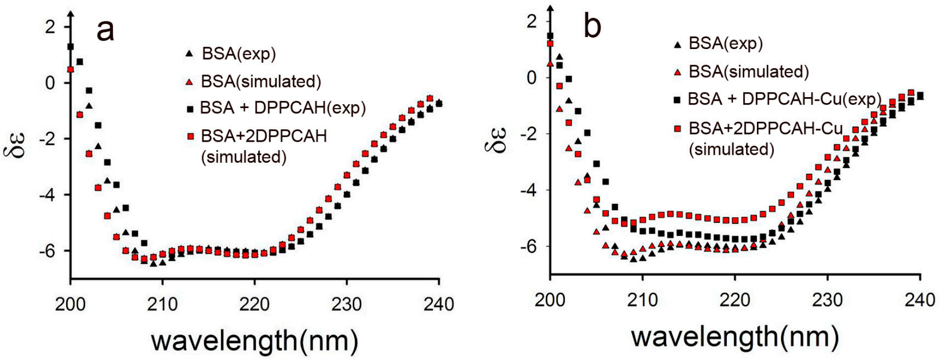

Figure S1. The simulated CD experimental data: (**a**) in the absence or presence of DPPCAH; (**b**) in the absence or presence of DPPCAH-Cu. The experimental and simulated as indicated in the figures.

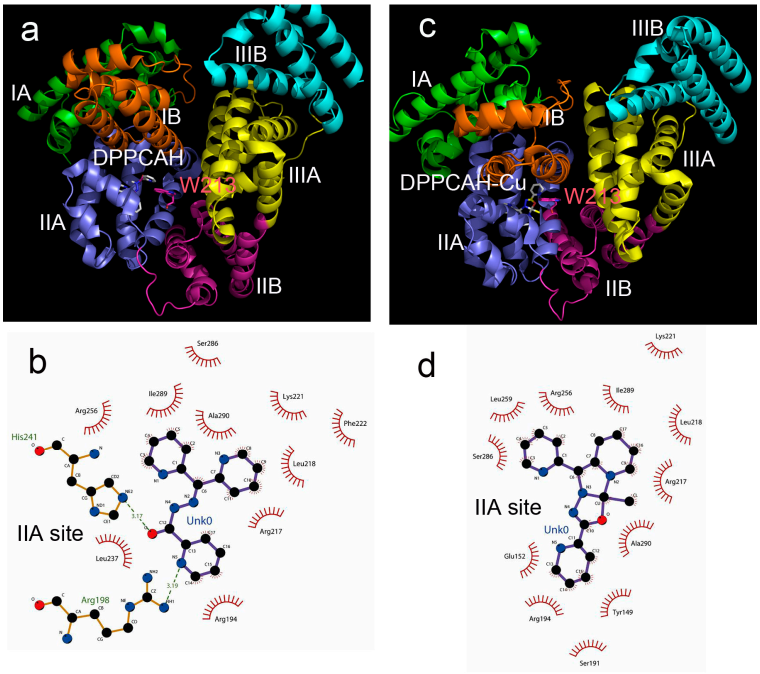

Figure S2. The interaction modes between BSA with DPPCAH and DPPCAH-Cu. (**a**) DPPCAH was located in IIA site; (**b**) DPPCAH interacted with environment residues (in the figure the Unk0 = DPPCAH); (**c**) DPPCAH-Cu was located in IIA site; (**d**) DPPCAH-Cu interacted with environment residues (in the figure the Unk0 = DPPCAH-Cu). Hydrogen bond was indicated by dark green dash lines (S2b,d). The eye-like curved dark red lines represented the hydrophobic contacted residues.
